# Supplementary material for: Polyketide synthase gene clusters in the ericoid mycorrhizal fungus Oidiodendron maius: genome-wide prediction, expression profiling, and functional characterization of knockout mutants
Source: Front Plant Sci. 2026 Sep 16;17:1924430. doi: 10.3389/fpls.2026.1924430 (PMC13623903; doi:10.3389/fpls.2026.1924430)
Supplement: Supplementary file 1 [file SupplementaryFile1.zip › Supplementary Materials/Supplementary Folder 1.zip!/Oidma1_AssemblyScaffolds_Repeatmasked/knownclusterblast/cluster37/jgi.p_Oidma1_170212_mibig_hits.html]

| MIBiG Protein | Description | MIBiG Cluster | MiBiG Product | % ID | % Coverage | BLAST Score | E-value |
| --- | --- | --- | --- | --- | --- | --- | --- |
| BAV19380.1 | NRPS-like\_enzyme | BGC0001390 | LL-Z1272beta\_biosynthetic\_gene\_cluster | 27 | 96.0915157293 | 289 | 1.7e-77 |
| AAS98174.1 | ochratoxin\_A\_non-ribosomal\_peptide\_synthetase | BGC0001030 | Ochratoxin\_A\_biosynthetic\_gene\_cluster | 27 | 65.490943756 | 252 | 2.2e-66 |
| AAG31130.1 | MxcG | BGC0001345 | Myxochelin\_biosynthetic\_gene\_cluster | 25 | 100.953288847 | 199 | 2.2e-50 |
| ADH01663.1 | putative\_polyketide\_synthase\_PKS3 | BGC0000099 | Monascorubrin\_biosynthetic\_gene\_cluster | 30 | 36.3203050524 | 156 | 2.2e-37 |
| ALI92655.1 | CitS\_citrinin\_polyketide\_synthase | BGC0001338 | Citrinin\_biosynthetic\_gene\_cluster | 31 | 36.7969494757 | 156 | 2.2e-37 |
| EED18001.1 | polyketide\_synthase,\_putative | BGC0000154 | Stipitatic\_acid\_biosynthetic\_gene\_cluster | 30 | 41.1820781697 | 155 | 4.9e-37 |
| AGN71604.1 | conidial\_yellow\_pigment\_biosynthesis\_polyketide\_synthase | BGC0000027 | Azaphilone\_biosynthetic\_gene\_cluster | 30 | 36.8922783603 | 139 | 1.6e-32 |
| CAP95404.1 |  | BGC0001404 | Sorbicillin\_biosynthetic\_gene\_cluster | 29 | 38.0362249762 | 136 | 2.3e-31 |
| AHJ31215.1 | Long-chain-fatty-acid--CoA\_ligase | BGC0000430 | Spumigin\_biosynthetic\_gene\_cluster | 24 | 44.3279313632 | 125 | 4.1e-28 |
| EHA28237.1 | hypothetical\_protein | BGC0001143 | Azanigerone\_biosynthetic\_gene\_cluster | 30 | 36.701620591 | 124 | 9.2e-28 |
| AAO23334.1 | NcpB | BGC0000397 | Nostocyclopeptide\_biosynthetic\_gene\_cluster | 24 | 58.627264061 | 118 | 5e-26 |
| AFO85453.1 | non-ribosomal\_peptide\_synthetase | BGC0000391 | Methylpendolmycin/pendolmycin\_biosynthetic\_gene\_cluster | 26 | 42.6120114395 | 118 | 6.6e-26 |
| AGJ76605.1 | HglB | BGC0000869 | Heterocyst\_glycolipids\_biosynthetic\_gene\_cluster | 26 | 41.2774070543 | 110 | 1.1e-23 |
| AAT12283.1 | LtxA | BGC0000384 | Lyngbyatoxin\_biosynthetic\_gene\_cluster | 27 | 39.9428026692 | 110 | 1.1e-23 |
| ALP32042.1 | CycB | BGC0001293 | Cyclizidine\_biosynthetic\_gene\_cluster | 26 | 40.4194470925 | 106 | 2e-22 |
| ABY83163.1 | Azi25 | BGC0000960 | Azinomycin\_B\_biosynthetic\_gene\_cluster | 25 | 54.9094375596 | 100 | 1.1e-20 |
| XP\_002384042.1 | NRPS-like\_enzyme,\_putative | BGC0001234 | 4,4'-piperazine-2,5-diyldimethyl-bis-phenol\_biosynthetic\_gene | 26 | 42.2306959009 | 96 | 1.6e-19 |
| BAP27942.1 | nonribosomal\_peptide\_synthetase | BGC0001085 | Teleocidin\_B\_biosynthetic\_gene\_cluster | 25 | 40.1334604385 | 94 | 6e-19 |
| BAT51067.1 | type\_I\_polyketide\_synthase | BGC0001296 | Streptazone\_E\_biosynthetic\_gene\_cluster | 27 | 42.0400381316 | 92 | 2.3e-18 |
| ctg1\_orf003 |  | BGC0000334 | Cyclosporin\_biosynthetic\_gene\_cluster | 23 | 51.1916110582 | 92 | 2.3e-18 |
| XP\_002384859.1 | NRPS-like\_enzyme,\_putative | BGC0001234\_c2 | 4,4'-piperazine-2,5-diyldimethyl-bis-phenol\_biosynthetic\_gene | 27 | 24.6901811249 | 89 | 2.5e-17 |
| EAW09117.1 | hybrid\_NRPS/PKS\_enzyme,\_putative | BGC0000983 | Cytochalasin\_biosynthetic\_gene\_cluster | 25 | 40.1334604385 | 82 | 3.1e-15 |
| EYT83459.1 | hypothetical\_protein | BGC0001213 | Nataxazole\_biosynthetic\_gene\_cluster | 25 | 31.1725452812 | 76 | 2.2e-13 |
| AHA12079.1 | polyketide\_synthase\_type\_1 | BGC0001172 | Chlorizidine\_A\_biosynthetic\_gene\_cluster | 29 | 24.4041944709 | 73 | 1.4e-12 |
| ABS90476.1 | NRPS | BGC0001106 | Oxazolomycin\_biosynthetic\_gene\_cluster | 23 | 56.3393708294 | 70 | 1.2e-11 |
| AGA37269.1 | NRPS | BGC0000819 | Paraherquamide\_biosynthetic\_gene\_cluster | 25 | 25.9294566254 | 69 | 2.7e-11 |
| AHZ34243.1 | CipF | BGC0001389 | Cichopeptin\_biosynthetic\_gene\_cluster | 23 | 45.757864633 | 69 | 3.5e-11 |
| AHZ34240.1 | CipC | BGC0001389 | Cichopeptin\_biosynthetic\_gene\_cluster | 24 | 46.3298379409 | 67 | 1.3e-10 |
| AGA37267.1 | NRPS | BGC0000816 | Malbrancheamide\_biosynthetic\_gene\_cluster | 26 | 23.832221163 | 65 | 3.9e-10 |
| AAY93354.1 | non-ribosomal\_peptide\_synthetase\_PvdD | BGC0000413 | Pyoverdine\_biosynthetic\_gene\_cluster | 21 | 56.9113441373 | 59 | 2.8e-08 |
| AGM16412.1 | paenibacterin\_synthetase\_A | BGC0000400 | Paenibacterin\_biosynthetic\_gene\_cluster | 21 | 62.5357483317 | 57 | 1.1e-07 |
| AAY93355.1 | non-ribosomal\_peptide\_synthetase\_PvdJ | BGC0000413 | Pyoverdine\_biosynthetic\_gene\_cluster | 21 | 60.3431839847 | 56 | 2.4e-07 |
| CAY48788.1 | putative\_non-ribosomal\_peptide\_synthetase | BGC0001312 | Viscosin\_biosynthetic\_gene\_cluster | 22 | 67.5881792183 | 55 | 5.2e-07 |
| AGM16414.1 | paenibacterin\_synthetase\_C | BGC0000400 | Paenibacterin\_biosynthetic\_gene\_cluster | 21 | 56.6253574833 | 55 | 5.2e-07 |
